# Prolonged inhibition of P-glycoprotein after exposure to chemotherapeutics increases cell mortality in multidrug resistant cultured cancer cells

**DOI:** 10.1371/journal.pone.0217940

**Published:** 2019-06-07

**Authors:** Amila K. Nanayakkara, Pia D. Vogel, John G. Wise

**Affiliations:** Center for Drug Discovery, Design and Delivery, The Center for Scientific Computing, and The Department of Biological Sciences, Southern Methodist University, Dallas, Texas, United States of America; University of Kansas Medical Center, UNITED STATES

## Abstract

One common reason for cancer chemotherapy failure is increased drug efflux catalyzed by membrane transporters with broad pump substrate specificities, which leads to resistances to a wide range of chemically unrelated drugs. This multidrug resistance (MDR) phenomenon results in failed therapies and poor patient prognoses. A common cause of MDR is over-expression of the P-glycoprotein (ABCB1/P-gp) transporter. We report here on an MDR modulator that is a small molecule inhibitor of P-glycoprotein, but is not a pump substrate for P-gp and we show for the first time that extended exposure of an MDR prostate cancer cell line to the inhibitor *following* treatment with chemotherapeutics and inhibitor resulted in trapping of the chemotherapeutics within the cancerous cells. This trapping led to decreased cell viability, survival, and motility, and increased indicators of apoptosis in the cancerous cells. In contrast, extended exposure of non-Pgp-overexpressing cells to the inhibitor during and after similar chemotherapy treatments did not lead to decreased cell viability and survival, indicating that toxicity of the chemotherapeutic was not increased by the inhibitor. Increases in efficacy in treating MDR cancer cells without increasing toxicity to normal cells by such extended inhibitor treatment might translate to increased clinical efficacy of chemotherapies if suitable inhibitors can be developed.

## Introduction

Chemotherapy treatments are often part of cancer therapies, either before surgery to decrease the size of existing tumors, or after surgery to target metastatic cells that may have migrated out of the primary site of the disease. For cancers that are not surgically accessible, chemotherapy is often the only treatment option. Some of these therapies can be remarkably effective, but unfortunately many cancers recur after initial, seemingly successful treatments and still others simply do not respond well to chemotherapies [1]. One common reason for the failure of chemotherapies is the expression of biochemical defense mechanisms in the cancer cells that have evolved to keep normal cells and tissues healthy. The phenomenon of multidrug resistances (MDR) in cancer chemotherapies is one such example, where certain members of the ABC transporter superfamily of membrane proteins [2], when expressed in cancerous cells, actively keep the cells free of the cytotoxic chemotherapeutics [3–8]. When expressed at high levels, proteins like P-glycoprotein (ABCB1, P-gp) [9], the breast cancer resistance protein (ABCG2, BCRP) [10], and/or the multidrug resistance associated protein 1 (ABCC1, MRP-1) [11], have the ability to remove most of the approved cancer chemotherapeutics from the cells, making chemotherapies ineffective.

In previous work from our group, we used computational methods to develop structural models of one of these pumps, P-gp,[12, 13] which were used in ultrahigh throughput *in silico* screening approaches to identify[14] and characterize [15, 16] drug-like compounds that inhibited P-gp and reversed multidrug resistance in several cancer cells in culture. The compounds were selected to inhibit P-glycoprotein by interfering with the transporter’s ability to utilize ATP to power drug efflux and to not be transport substrates of the pump. These inhibitors have been shown to resensitize MDR cancer cells in culture and to enhance the killing of MDR cancer cells in 3-dimensional microtumor spheroids[15, 16]. Most of the inhibitors of P-gp that were assessed previously were transport substrates of the pump [6, 17–19]. The P-gp inhibitors identified in [14] were found to not be transported out of cells by the transporter[16] as was the original premise of the computational search employed[14]. This characteristic is viewed as an important improvement over previous generations of P-gp inhibitors. Active removal of P-gp inhibitors from the cells likely requires overall higher extracellular concentrations for efficacy, causing off-target toxicities once the compounds are geared towards clinical applications as co-therapeutics to treat chemotherapy insensitive cancers.

We show here in a multidrug resistant cancer cell line that over-expresses P-gp, that the continued presence of an inhibitor of P-glycoprotein after a short exposure of the cells to chemotherapeutic in the presence of the inhibitor, and the subsequent removal of the chemotherapeutic from the medium in the presence of the inhibitor, significantly increases the effectiveness of the therapy. We have shown here that this “extended P-gp inhibitor” treatment correlated with significantly increased cellular retention of chemotherapeutic, reduced cancer cell viabilities, reduced cancer cell migration, and increased morphological indicators of apoptosis and cancer cell mortality, thereby demonstrating the increased efficacy of the treatment. In isogenic cancer cells with low expression of P-gp, no increases in toxicity and associated effects from this "extended P-gp inhibition" were observed, so the observed effects are target (P-glycoprotein) specific.

We have explored these effects with one of the P-glycoprotein inhibitors previously identified by us [14–16], but it is likely that these effects will be generalizable and work with other P-gp inhibitors as well. Our results suggest that the efficacy of chemotherapeutics in killing cancerous cells can be extended beyond the actual treatment with the chemotherapeutic drug and after the chemotherapeutic was removed from the culture medium. These results have implications on the potential benefits of ABC transporter inhibitors in chemotherapy treatment of multidrug resistant cancers. We believe these results are important findings that may eventually increase the efficacy of treating MDR cancers while simultaneously decreasing exposure of normal cells to toxic chemotherapeutics.

## Materials and methods

### Cell lines and cell culture

The drug sensitive DU145 human prostate cancer cells [20] as well as the multidrug resistant sub-line, DU145TXR [21] were generous gifts from Dr. Evan Keller (University of Michigan, Ann Arbor, MI). The MDR DU145TXR cell line was maintained under positive selection pressure by supplementing complete medium with 10 nM paclitaxel (“PTX”). The cell lines were maintained in complete media consisting of RPMI-1640 with L-glutamine, 10% fetal bovine serum (FBS; BioWest, Logan, UT or Corning, NY), 100 U/mL penicillin and 100 μg/mL streptomycin in a humidified incubator at 37°C and 5% CO_2_. Cell culture materials were purchased from Corning Inc. (Corning, NY) unless otherwise stated. The chemotherapeutics paclitaxel and vinblastine (“VIN”) were purchased from Acros Organics, NJ, and MP Biomedicals, France, respectively. Human lung fibroblast cells, HFL-1 cells[22], were maintained in complete medium consisting of F12K with L-glutamine, 10% fetal bovine serum, 100 U/mL penicillin and 100 μg/mL streptomycin in a humidified incubator at 37°C and 5% CO_2_.

The cell lines used in this work were analyzed for expression of P-glycoprotein using q-PCR and Western blot analyses. Experiments using q-PCR and Western blot techniques confirmed that DU145TXR markedly over-expresses P-glycoprotein compared to its parental line, DU145. Additional q-PCR analyses indicated the absence of any observable mycoplasma contamination.

### Daunorubicin accumulation and release

**Fig 1:**
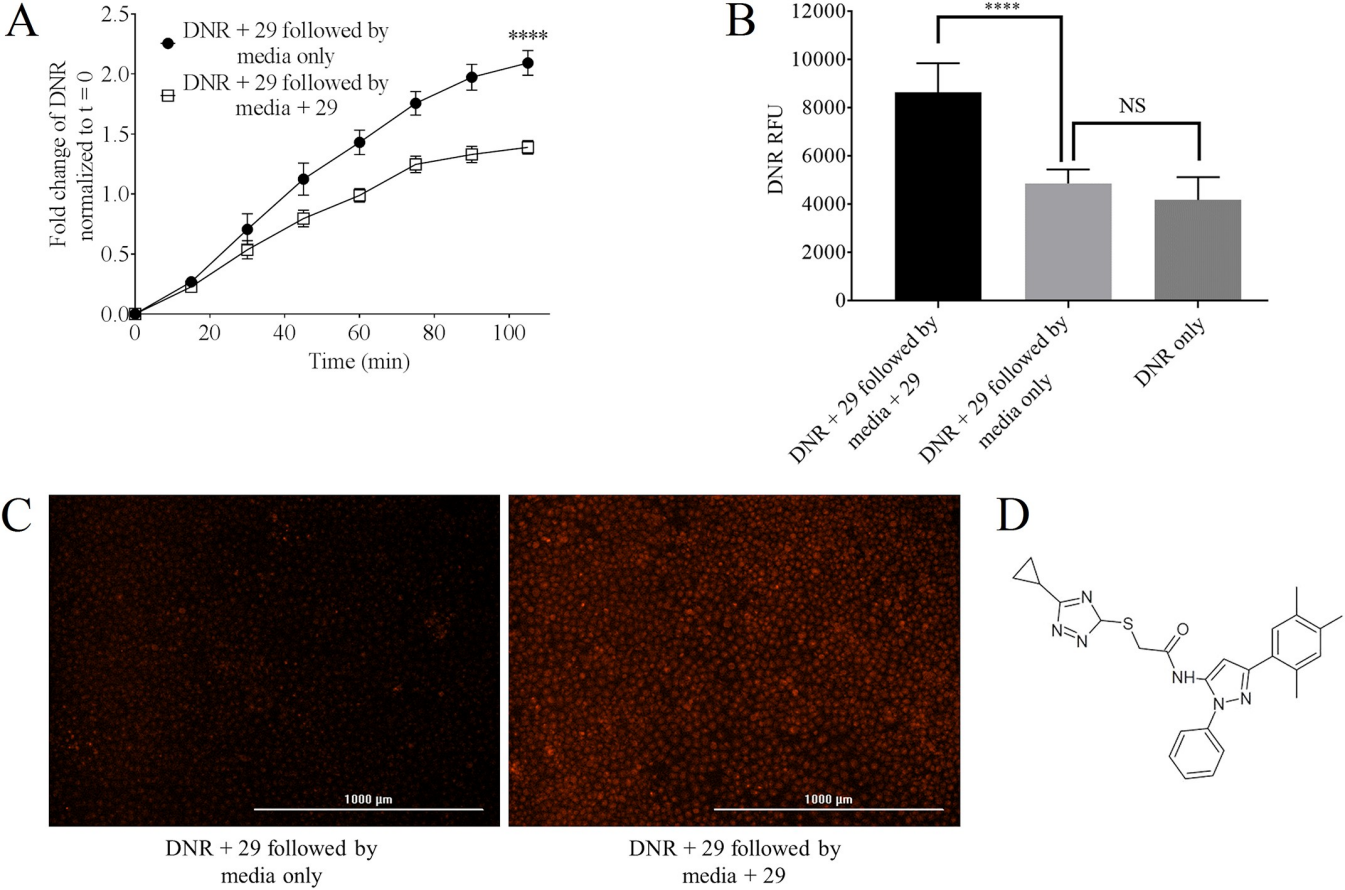
Effects of P-gp inhibition on the retention of daunorubicin in the multidrug resistant prostate cancer cell line, DU145TXR. **Panel A**–Fluorescence of daunorubicin released from the cells in the presence or absence of P-gp inhibitor **29** was measured over time and results were normalized to t = 0. In these experiments, cells were preloaded with daunorubicin by incubation in the presence of the P-gp inhibitor **29** as described in Methods. After the preloading, cells were washed and media were replaced with complete media with or without **29**. Results indicate that the presence of P-gp inhibitor in the medium decreased the relative rate of daunorubicin release. Data are expressed as average ± S.D. of duplicate experiments (n = 12; **** P < 0.0001). **Panel B**–The total fluorescence of daunorubicin accumulated in the cells was measured at the 105 min time point as indicated in **A** after washing and complete lysis of the cells as described in Methods. A significant amount of daunorubicin was retained in the cells in the presence of P-gp inhibitor **29** as opposed to those cells maintained in its absence. The upper right bar graph shows the quantity of daunorubicin accumulated in cells that were exposed to daunorubicin for the entire time-course in the absence of P-gp inhibitor. The figure indicates significantly decreased steady state accumulation of chemotherapeutic in the absence P-gp inhibition compared to that observed in the continued presence of inhibitor. Data are expressed as average ± S.D. of duplicate experiments (n = 12; **** P < 0.0001; “NS” = no significant difference). **Panel C**–Fluorescence images of daunorubicin retention in the DU145TXR cells at the 105 min time point. After washing the cells with cold PBS as described in Methods, fluorescence micrographs were obtained using a Cytation 5 imager and a Texas Red filter. The concentration of compound **29** was 25 μM and daunorubicin was 10 μM. **Panel D**–Structure of compound **29**.

DU145TXR cells were seeded in 96 wells plates at 3000 cells per well in complete media and allowed to grow until confluence. After removal of medium, the cells were treated with 25 μM P-gp inhibitory compound **29** [14] (IUPAC: 2-[(5-cyclopropyl-4H-1,2,4-triazol-3-yl)sulfanyl]-N-[2-phenyl-5-(2,4,5-trimethylphenyl)-pyrazol-3-yl; SMILES: Cc5cc(c3cc(NC (= O)CSC1N = C(N = N1)C2CC2)n(n3)c4ccccc4)c(C)cc5C, Fig 1D) and 10 μM daunorubicin (“DNR”, MP Biomedicals, France) diluted into complete medium. After 1.5 hours of incubation, medium was removed and cells were washed three times with 200 μL of cold PBS (137 mM NaCl, 2.7 mM KCl, 10 mM Na_2_HPO_4_, 1.8 mM KH_2_PO_4_, pH 7.4). Half of the test wells were then incubated with 25 μM P-gp inhibitor **29** diluted into phenol red free RPMI-1640 and the other half of the test wells were supplemented with phenol red free RPMI-1640 containing 0.5% DMSO (vehicle). The fluorescence of daunorubicin released from the cells was measured over 105 minutes in 15-minute intervals at excitation at 488/20 nm and emission at 575/20 nm using a Cytation 5 imaging multi-mode reader (BioTek, Winooski, VT).

After 105-minutes, the amount of daunorubicin remaining in the cells was measured qualitatively and quantitatively. To qualitatively observe the daunorubicin retained in the cells, medium was removed and the cells were fixed with 4% para-formaldehyde in PBS. The cells were imaged using a Cytation 5 imaging multi-mode reader with a Texas red fluorescence filter. To quantify the daunorubicin that had remained in the cells, medium was removed from each well and cells were lysed in 100 μL of PBS containing 0.5% SDS and 0.5% Triton X100. The fluorescence of daunorubicin was measured using the Cytation 5 imaging multi-mode reader.

### Resazurin cell viability assays

**Fig 2:**
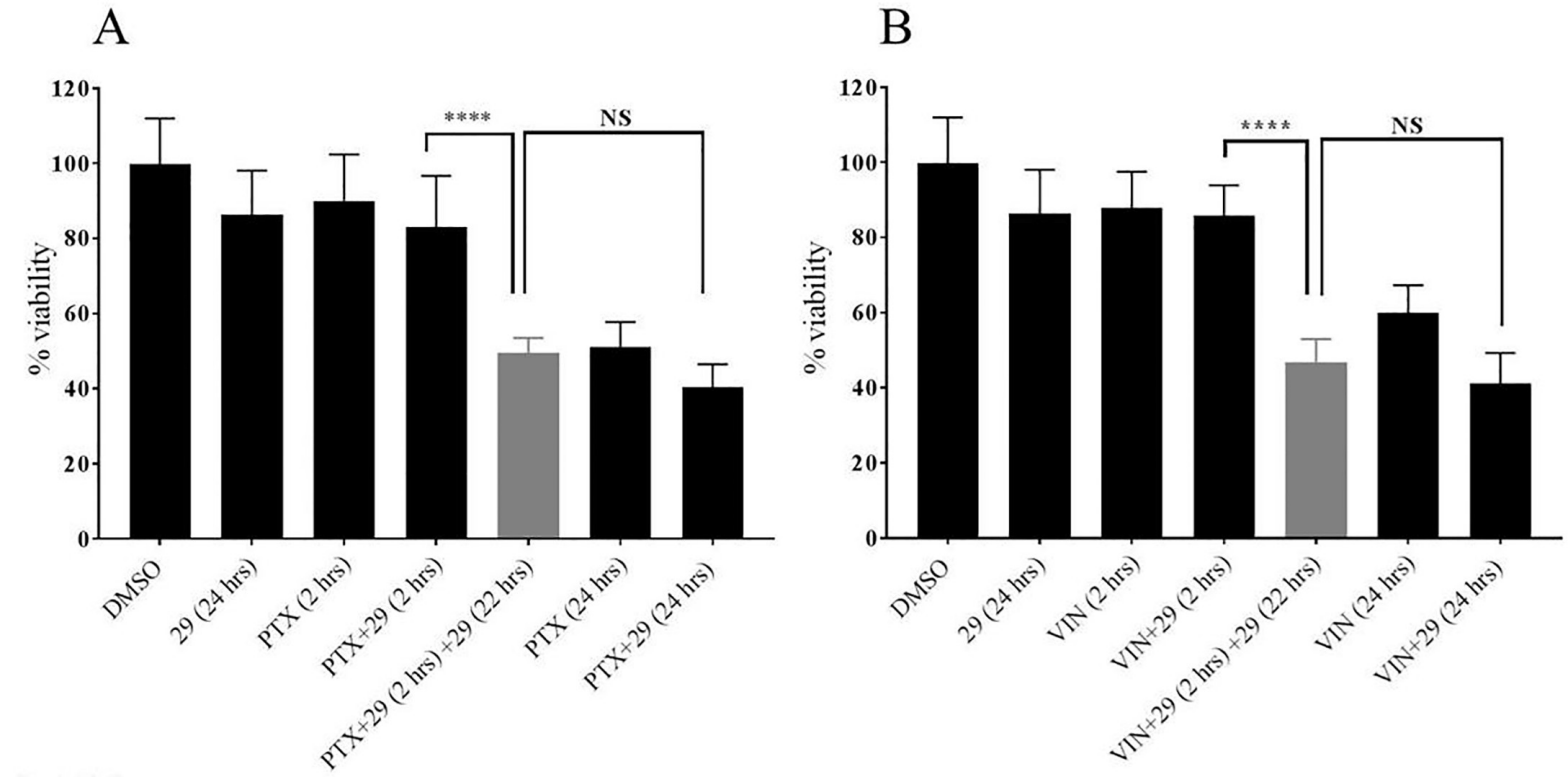
The increased chemotherapeutic retention in MDR cells treated for extended times with P-glycoprotein inhibitor 29 decreased viabilities of DU145TXR cells. In these experiments, DU145TXR cells were preloaded with either paclitaxel (**A**) or vinblastine (**B**) in the presence of P-gp inhibitor **29** unless indicated in the figure. After the preloading incubation, cells were washed and media were replaced with complete media with the indicated additions. Control experiments included exposure of the cells to DMSO alone (0.5% final concentration), P-gp inhibitor **29** alone (25 μM), paclitaxel (PTX, 10 μM), vinblastine (VIN, 10 μM) or the combinations and times indicated. The percentage of viable cells present at the end of the experiment is expressed as percentages of the DMSO-vehicle control experiments. Data are expressed as averages +/- S.D. of duplicate experiments (n = 8; **** p < 0.0001). “NS” = not significantly different. Measurements were made as described in Methods using a Cytation 5.

Cells were trypsinized from monolayers and seeded into a 96 well plate with 3000 cells in 150 μL of complete media per well. After 48 hours of incubation, cells were treated for 2 hours with chemotherapeutics and / or P-gp inhibitor **29** dissolved in DMSO, and / or DMSO alone diluted into complete medium. After 2 hours, cells were washed with cold PBS that did not contain chemotherapeutic and treatment was continued for an additional 22 hours with compound **29** alone to study the effect of retaining paclitaxel and vinblastine by compound 29. For the control experiment cells were treated with inhibitor free media for additional 22 hours after initial treatment. Further control experiments were included as indicated in Fig 2. At 18 hours of treatment, 440 μM of resazurin (Acros Organics, NJ) solution prepared in PBS was added for viability assays as described in [23]. The resulting resafurin fluorescence was measured after 6 hours of incubation of the cells with resazurin by excitation at 530 nm and emission at 590 nm using a Cytation 5. The percent viability was calculated using the DMSO treated cells as representative for 100% viability. Background fluorescence was determined using resazurin and complete medium without cells.

$$\%\text{Viability}\;=\;\frac{\text{Fluorescence}\;\text{of}\;\text{experimental}\;\text{cells}-\text{Background}\;\text{fluorescence}*100}{\text{Fluorescence}\;\text{of}\;\text{DMSO}\;\text{treated}\;\text{cells}-\text{Background}\;\text{fluorescence}}$$

The results from these assays were plotted as the mean with standard deviation (SD) of at least eight replicates per treatment from two independent experiments using the program GraphPad Prism (La Jolla California, USA, Version 7).

### Colony formation assays

Colony formation assays were performed similar to [24] with slight modifications. DU145 TXR cells were seeded in 24 well plates with 250 cells per well. After 24 hours, cells were treated for 2 hours with paclitaxel and / or P-gp inhibitor, compound **29**, dissolved in DMSO, or DMSO controls diluted in complete medium. The concentration of **29** and paclitaxel was 10 μM. The cells were then washed with cold PBS and exposed to compound **29** alone (“extended **29** treatment”) for another 22 hours. Control experiments were performed as described in viability assays. The medium was then removed and cells were allowed to form colonies for 5 days in drug- and compound **29**-free complete media. To visualize the colonies, the medium was removed and cells were fixed with a mixture of methanol and acetic acid 3:1 (v/v) solution. After 5 minutes, the fixation solution was removed and the cells were stained with 0.5% w/v crystal violet (Alfar Aesar, MA) in 25% methanol for 30 minutes. After removal of crystal violet, the whole plates were washed with running water to remove excess dye. Colonies visible to the naked eye were counted and recorded by persons blinded to all experimental conditions. The number of colonies were normalized to vehicle-treated controls as percentages. The experiment was repeated two times with a total of three replicates each.

### Scratch assays

Scratch assays were performed as outlined previously [25] with minor modifications. Cells were trypsinized from monolayers and diluted in complete culture medium to a density of 45,000 cells in 400 μL cell suspension per well in 48-well plates and cultured until fully confluent. The monolayers of cells were then scratched using a 200 μL pipette tip. The medium was removed and the cells were washed with PBS to remove floating cells. Immediately after the scratching and media addition, the wounds were imaged using the Cytation 5. Complete medium with low serum content (1% v/v) was then added to the wells together with 0.5 μM paclitaxel with or without 15 μM P-gp inhibitor, compound **29**. In the no-treatment control only 0.5% final volume of DMSO was added in place of drugs and/or P-gp inhibitor. After 1 hour incubation, medium was removed from all the wells and the cells were washed with cold PBS. All test wells were incubated with 0.5% DMSO containing media with 1% FBS except for the wells that contained compound **29** only, and the wells with extended compound **29** exposure after co-treatment with paclitaxel and compound **29**. Extended treatment of P-gp inhibitor **29** was carried out in a concentration of 7.5 μM for 13 hours. The “wounds” in the confluent cell layers were then re-imaged and the area of the wounds before and after each treatment were quantified using ImageJ software [26]. The percentage of wound closure in each test was calculated compared to vehicle treated experiments. Each individual experiment was performed in triplicate and 2 images were obtained for each well. The whole experiment was repeated at least once, and n = 12 was used for the statistical analysis.

### Fluorescence microscopic analysis of cell apoptosis

Double staining with acridine orange/ ethidium bromide (AO/EB) is a reliable method to detect apoptosis and was carried out as described in [27] with slight modifications. 16,000 cells were seeded in 48 well plates in 300 μL of complete media and incubated for 48 hours. After 48 hours, cells were treated for 1 hour with 10 μM paclitaxel and 15 μM P-gp inhibitory compound **29** in DMSO or DMSO controls. Cells were then washed with cold PBS and an “extended treatment” with compound **29** alone was carried out as described above for another 23 hours. Cells not treated with compound **29** were treated with 0.5% DMSO (vehicle). The AO/EB dual stain containing solution (100 μg/ml each) was then added to each well and images were acquired using a Cytation 5 with GFP (for green fluorescence from acridine orange) and Texas red fluorescence (for red fluorescence from ethidium bromide).

### Statistical analysis

Statistical differences were determined by using t test in program GraphPad Prism (La Jolla California, USA, Version 7). Significance of results is as described in individual figure legends.

### Data availability

The observed data that was used to create the Figures presented here is available in the Supporting Information file associated with this manuscript.

## Results

### The presence of P-gp inhibitor 29 results in cellular retention of previously accumulated daunorubicin in multidrug resistant prostate cancer cells

In experiments aimed at investigating whether the rate of release of chemotherapeutic drugs from multidrug resistant human prostate cancer cells was affected by the continued presence of the P-gp inhibitor, compound **29** [14, 15] (Fig 1D), we first exposed DU145TXR cells [21] to daunorubicin in the presence of compound **29** to enable the cells to significantly accumulate the chemotherapeutic. The media containing daunorubicin and compound **29** were then removed and replaced with fresh media with or without addition of **29**. The relative rates of release of daunorubicin from the cells were observed using the intrinsic fluorescence of the chemotherapeutic. We used a relatively high concentration of inhibitor **29** in these assays (25 μM) to insure that maximal inhibition of P-glycoprotein was achieved. We have previously seen that we can completely reverse P-gp mediated MDR in these cells at this concentration. It should be noted that inhibitor **29** showed significant inhibition of P-gp and reversal of MDR at concentrations lower than 25 μM [15, 16].

Fig 1A shows a time-course of release of daunorubicin over 105 minutes in the absence and presence of added P-gp inhibitor. The figure clearly shows that daunorubicin release was significantly slower in the presence of **29** than it was in its absence. Fig 1B shows the relative amounts of daunorubicin retained by these cells at the end of the 105 min time-course. After washing the cells in cold PBS at the 105 minute time point of the daunorubicin release assay, these cells were lysed as described in Methods and the total relative fluorescence of each well was measured using the Cytation 5. It is clear that the MDR prostate cancer cells retained significantly more daunorubicin when incubated in the presence of inhibitor **29** than cells incubated without P-gp inhibitor for the same period of time (Fig 1B, compare black bar to light gray bar). When DU145TXR cells were incubated with daunorubicin in the presence of **29** followed by a release period in the absence of **29**, the overall retention of daunorubicin was comparable to experiments where cells were incubated with daunorubicin alone (Fig 1B, compare light gray to dark gray bars). This indicates that the cells that were loaded with daunorubicin in the presence of **29** released all of the daunorubicin accumulated due to P-gp inhibition within a relatively short time period (~105 min) when P-gp inhibition was not maintained. The data also suggest that the intracellular steady state concentration of chemotherapeutic reached in the presence of P-gp inhibitor far exceeded the intracellular steady state concentration of therapeutic when P-gp was not inhibited. The fluorescence micrographs presented in Fig 1C of cells preloaded with daunorubicin plus **29** followed by 105 min of daunorubicin release in the presence (right panel) or absence (left panel) of P-gp inhibitor **29** demonstrate qualitatively the higher retention of daunorubicin observed in these MDR prostate cancer cells when P-gp inhibition was maintained after chemotherapeutic treatment ended.

### The viability of multidrug resistant, P-gp overexpressing prostate cancer cells is reduced by extended exposure to P-gp inhibitor 29 after exposure to chemotherapeutics

As shown in Fig 1, increased amounts of chemotherapeutics were retained in P-gp-overexpressing cells when the cells were continuously exposed to P-gp inhibitor **29** after removal of chemotherapeutic from media. In order to determine if this increased retention of chemotherapeutics in the cells in the extended presence of **29** resulted in decreased cell viabilities, we carried out a series of resazurin viability experiments[23] as modified in [16] using the same concentration of **29** (25 μM) used in Fig 1. In these assays, we first exposed DU145TXR cells to paclitaxel (Fig 2A) or vinblastine (Fig 2B) in the presence of P-gp inhibitor **29** and allowed the cells to accumulate the chemotherapeutics as described for daunorubicin in Fig 1. After a 2-hour pre-incubation in the presence of inhibitor and chemotherapeutic, the media containing chemotherapeutic and inhibitor were removed and replaced by complete media with or without P-gp inhibitor **29**. This was followed by a 22 hour incubation period, resulting in total incubation times of 24 hours. Control groups were maintained with drug free complete media (0.5% DMSO). It can be seen in Fig 2 that treatment with the P-gp inhibitor alone for 24 hours did not significantly affect cell viability as judged by the resazurin assay. Addition of paclitaxel (Fig 2A) or vinblastine (Fig 2B) alone for 2 hours, or paclitaxel or vinblastine plus inhibitor **29** for two hours also did not significantly decrease viability of these MDR prostate cancer cells when compared to the DMSO-vehicle controls. In stark contrast however, was treatment of the cells with either paclitaxel or vinblastine in the presence of P-gp inhibitor **29** for two hours, followed by an extended P-gp inhibitor exposure for 22 hours after washing the cells free from non-internalized chemotherapeutic. This extended inhibitor treatment resulted in significant reduction in cell viability for both paclitaxel and vinblastine (gray bars in Fig 2A and 2B). The decreased viability of the MDR cancer cells upon “extended P-gp inhibitor” treatment was not significantly different than 24 hour continuous exposures to either paclitaxel or vinblastine alone with or without P-gp inhibitors present (Fig 2A and 2B, rightmost three bars). These results indicate that 2 hour exposures to chemotherapeutics combined with a 24 hour total exposure to P-gp inhibitors decreased the viability of these MDR cancer cells equally well as compared to a continuous 24 hour treatment with chemotherapeutic.

### Survival of P-gp overexpressing MDR prostate cancer cells is reduced by extended exposure to P-gp inhibitor 29 after exposure to chemotherapeutics

**Fig 3:**
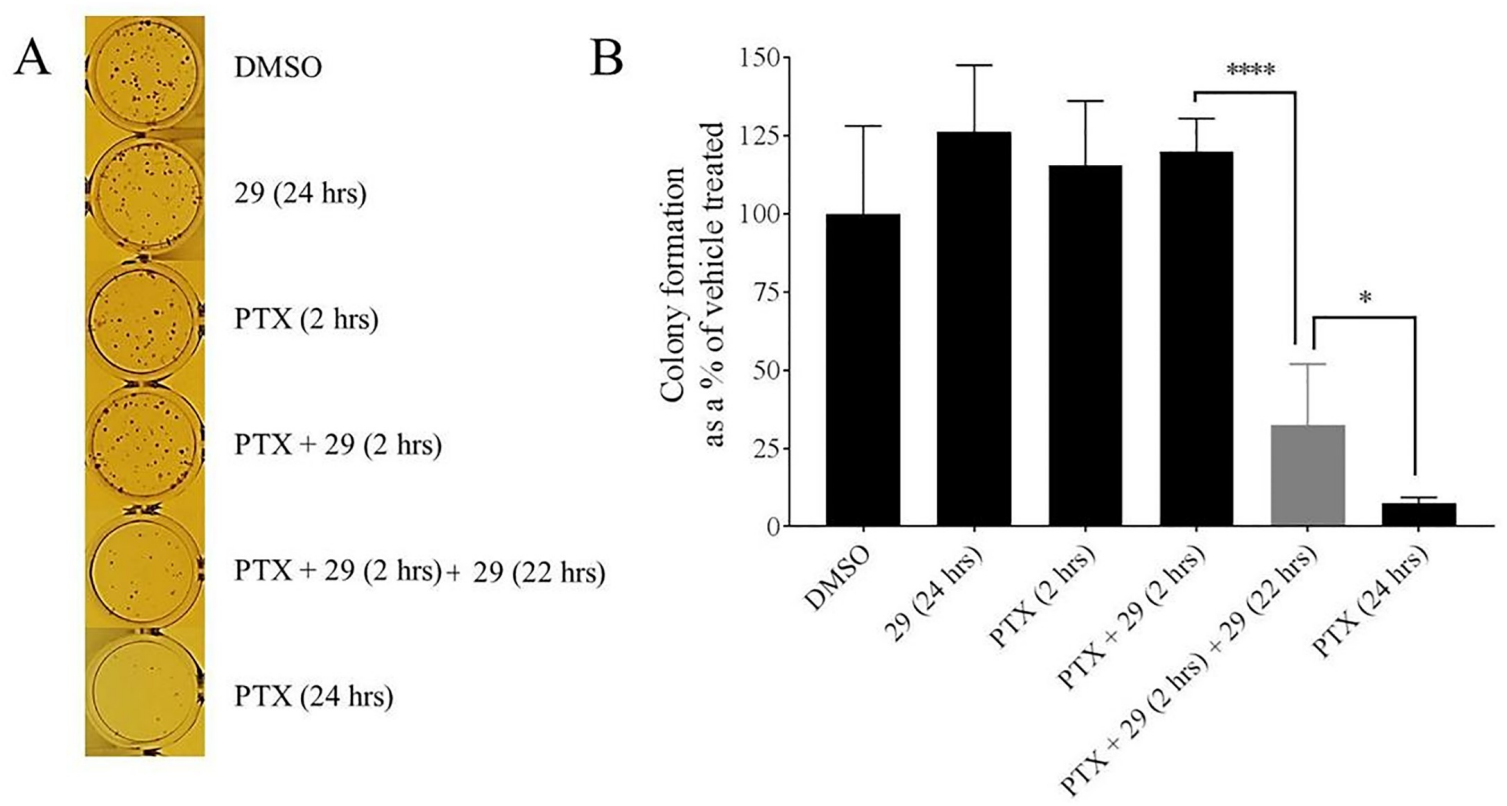
The increased chemotherapeutic retention in MDR cells treated for extended times with P-glycoprotein inhibitor 29 decreased cell survival as assessed by colony formation assays. Experiments were performed similarly to those reported in Fig 2 except that treatments with chemotherapeutic and / or inhibitor **29** were followed by a five day recovery period in complete media without addition of chemotherapeutics or P-gp inhibitor. The chemotherapeutic in these experiments was paclitaxel (PTX). At the end of the five day incubation, cells were fixed and stained. Colony formation was assessed as described in Methods. **A**—Representative images of the crystal violet stained colonies that were counted for the quantification shown in panel B. **B**–Quantification of the number of colonies observed after treatments with the indicated compounds and times. The data were normalized to the DMSO only control experiments and are expressed as average ± S.D. of duplicate experiments (n = 6; **** P < 0.0001; * < 0.1). The concentration of **29** and paclitaxel was 10 μM.

Fig 2 demonstrated that increased retention of chemotherapeutics due to extended P-gp inhibitor treatment led to decreased cellular viabilities. Although the resazurin assay is known to respond to mitochondrial metabolism, and this in turn is correlated with cellular viability [28, 29], it was of interest to assess whether these extended P-gp inhibitor treatments after short chemotherapy exposures actually decreased cancer cell survival. With this aim, we performed colony formation experiments in a manner similar to the viability experiments shown above. In these experiments, the cells were treated with the indicated compounds (paclitaxel (10 μM) and/ or P-gp inhibitor (10 μM)), were then washed and subsequently incubated for five days in the absence of added chemotherapeutic and P-gp-inhibitor **29**. At the end of the five-day period, cells were fixed and stained, and the colonies were counted by individuals with no knowledge of the experimental details to avoid any obvious biases. Fig 3A shows representative images of crystal violet stained colonies visible to the naked eye at the end of the experiment. Fig 3B shows that the observed colony numbers as shown as a percentage of the DMSO-only control were similar to the results reported in the viability assays in Fig 2. No significant difference in cell survival was observed when the cells were exposed to P-gp inhibitor **29** for 24 hours, or 2 hour exposure to paclitaxel, or 2 hour exposure to paclitaxel and inhibitor **29**. In contrast, and similar to the viability results presented in Fig 2, co-treatment of these MDR cancer cells for 2 hours with paclitaxel and P-gp inhibitor **29**, followed by extended treatment for 22 hours with only compound **29** resulted in a very significant decrease in cellular survival, which was only slightly surpassed by a 24 hour continuous exposure of the cells to paclitaxel (Fig 3B, rightmost three bars).

### Increased retention of chemotherapeutic in MDR cancer cells treated for extended times with P-gp inhibitor 29 reduced cell migration

**Fig 4:**
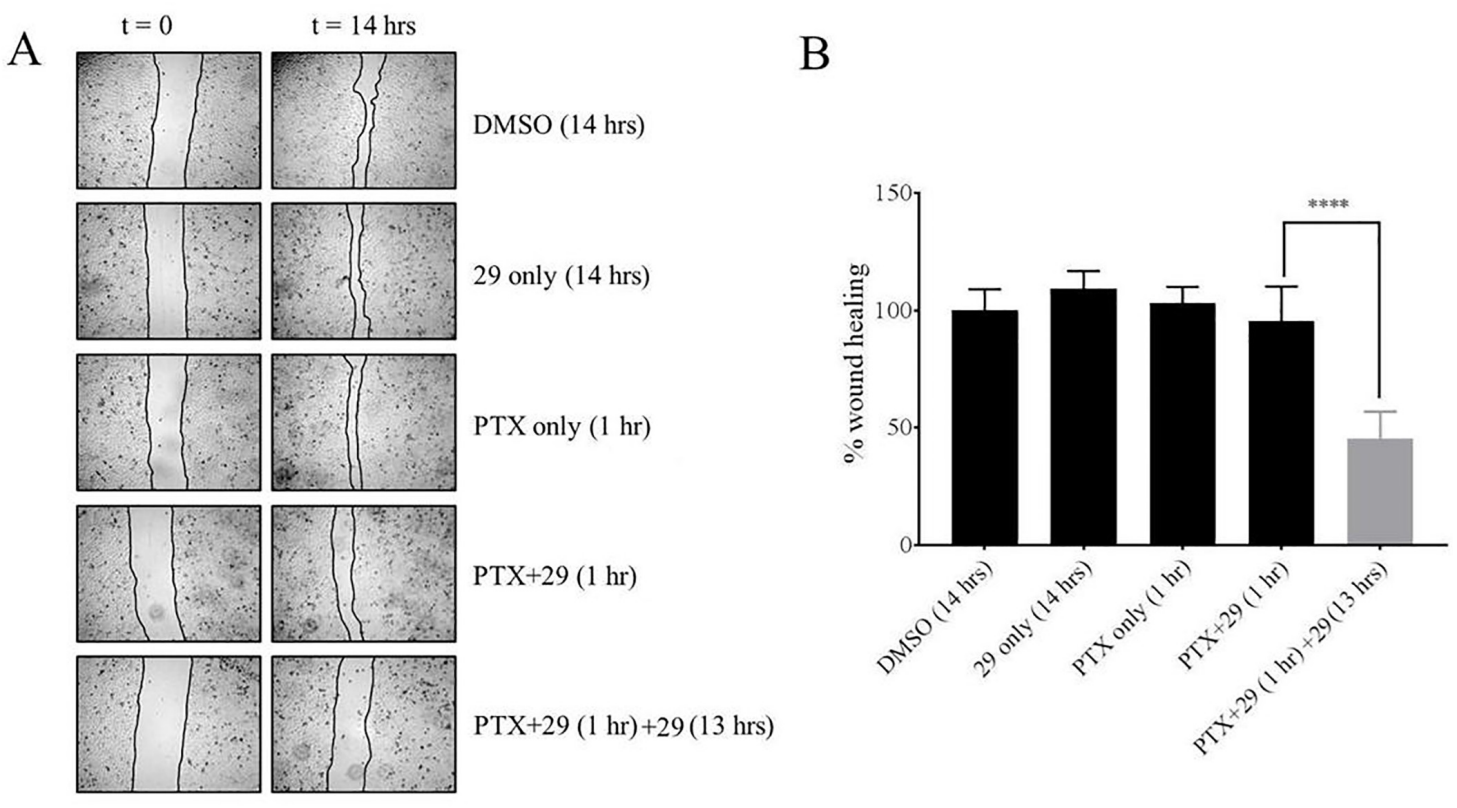
Increased retention of chemotherapeutics in MDR cells treated for extended times with P-glycoprotein inhibitor decreases cell motility as determined by wound healing assays. The treatments of cells with chemotherapeutic and P-gp inhibitor **29** (as indicated) were performed similarly to those reported in Figs 1–3 except that these assays were designed to detect cellular migration independent of proliferation. **A**—Typical bright field images at zero time and after 14 hours for the chemotherapeutic and P-gp inhibitor treatments as indicated in the Figure. Microscopy was as described in Methods using a Cytation 5 imager at 4 x magnification. The concentration of **29** was 15 μM for the initial treatment with 0.5 μM paclitaxel as indicated. These concentrations were chosen to show the migration effects without significant cell death. Extended treatment of P-gp inhibitor **29** was carried out in at 7.5 μM for 13 hours. **B**–Results of wound healing assays are reported as a percentage of closure of the wound after 14 hours relative to the DMSO vehicle-only controls. Cells were treated with P-gp inhibitor **29** and paclitaxel for the times indicated. Data are expressed as average ± S.D. of duplicate experiments (n = 12; **** P < 0.0001).

Wound healing assays [16, 25, 30, 31] have been used to detect and quantify the ability of cells to migrate from confluent to non-confluent growth areas. These assays use media containing low concentrations of serum to inhibit cellular proliferation, but these suboptimal growth conditions do not inhibit the abilities of cells to migrate into non-confluent areas on a culture plate. In addition, relatively short treatment times of maximally 14 hours were used to ensure that cellular proliferation was limited. To assess whether the increased retention of chemotherapeutics during extended treatment of MDR cancer cells with P-gp inhibitor **29** would affect cancer cell migration, wound healing assays were performed as described in Methods using DU145TXR cells. We used a relatively low concentration of paclitaxel (0.5 μM) in these assays so that significant cell death during the course of the experiments did not occur. The results shown in Fig 4A and 4B suggest that 14 hour treatments with inhibitor **29** alone or a one hour treatment with paclitaxel alone did not affect cancer cell migration relative to the vehicle-only DMSO control. A one-hour treatment of the cells with a combination of paclitaxel and P-gp inhibitor **29** likewise did not affect cell migration. In contrast, a short 1-hour co-treatment with chemotherapeutic and compound **29**, followed by a 13-hour exposure to P-gp inhibitor **29** reduced cell migration by nearly 50%. The cultures used in this assay were observed to be quite sensitive to paclitaxel, most likely because of the minimal FBS supplements used to keep cells from proliferating. Because of this sensitivity, we were not able to report on cell migration in a 14 h paclitaxel only control since a significant amount of cell death occurred under the conditions of this assay (data not shown).

Similar to the results shown in Figs 2 and 3, the experiments shown in Fig 4 also clearly demonstrated that extended exposure to the P-gp inhibitor after relatively short exposures to chemotherapeutics dramatically enhanced the effects of the drugs on the cancer cells. In the case of the migration assays, it should be noted that the main mode of action of paclitaxel is blocking microtubule disassembly processes which are required for the cytoskeletal remodeling necessary for effective cell mobility. Decreased cell mobility is therefore consistent with continued presence of paclitaxel in the cells during treatment with the P-gp inhibitor.

### Cellular retention of chemotherapeutics during extended exposure to inhibitor 29 increases apoptosis in P-gp over-expressing DU145TXR cells

**Fig 5:**
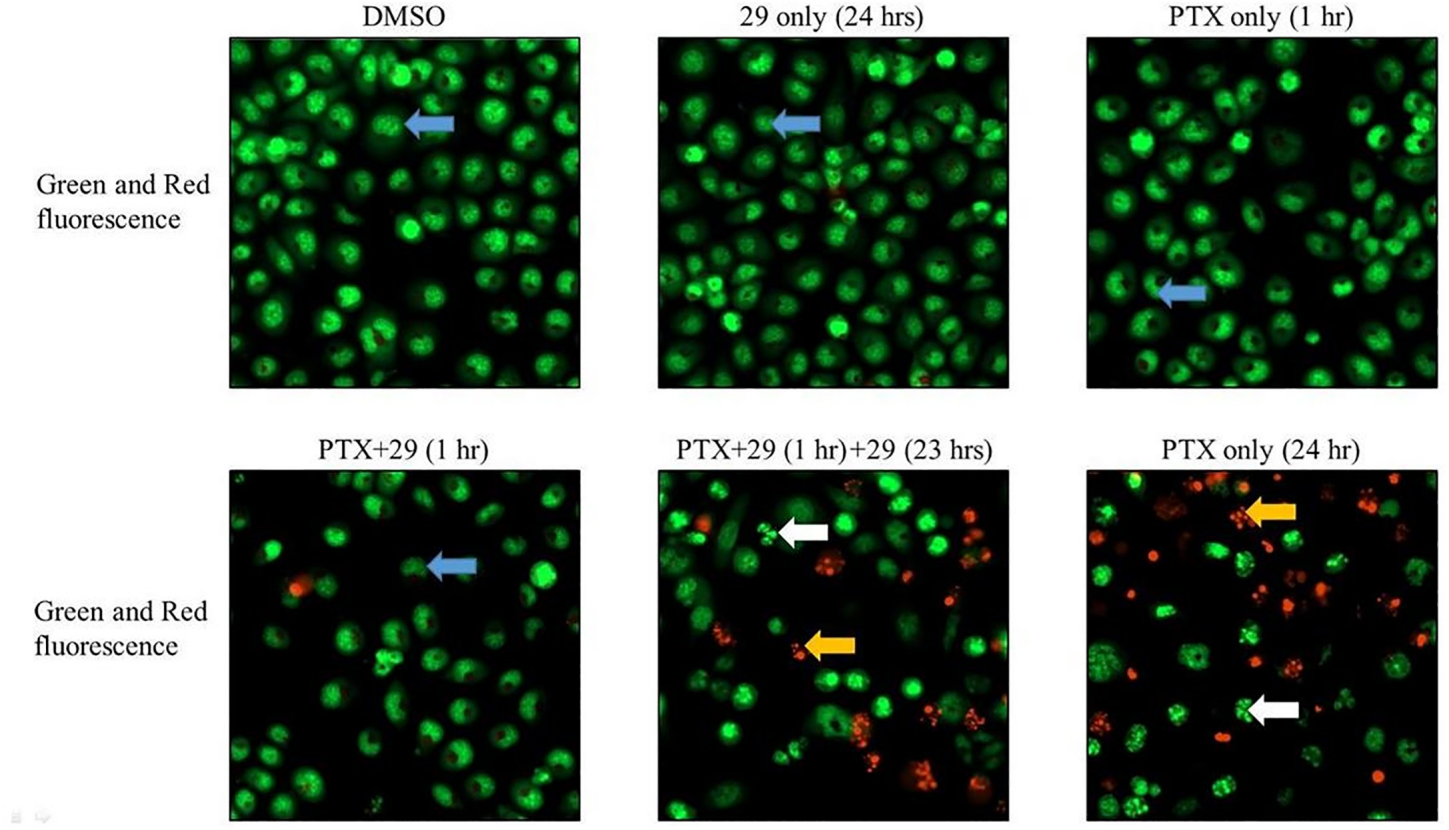
Increased apoptosis in P-gp over-expressing cancer cells after chemotherapeutic exposure when followed by extended P-gp inhibitor treatment. P-gp over-expressing prostate cancer cells, DU145TXR, were exposed to 10 μM paclitaxel with or without 15 μM P-gp inhibitor **29** for 1 hour with or without extended P-gp inhibitor **29** treatment for another 23 hours as indicated in the figure. Cells were exposed to both acridine orange and ethidium bromide after the treatments were concluded. Blue arrows identify cells with highly organized nuclear morphologies typical of non-apoptotic cells. White arrows show cells with nuclear—chromatin fragmentation typical of apoptotic cells. Yellow arrows show dead cells with fragmented nuclei. Fluorescent images were obtained as described in Methods using a Cytation 5 imager.

Although Fig 2 indicated that increased and prolonged retention of chemotherapeutics in the presence of P-gp inhibitor **29** reduced metabolic activity and although the results of colony formation assays in Fig 3 indicated a significant decrease in the survival of cancer cells after such extended inhibitor treatments, these results did not directly demonstrate increased cell mortality *via* apoptotic mechanisms. To assess cell death mechanism, the cancer cells were exposed to both acridine orange and ethidium bromide to attempt to discern early and late morphological changes to cells undergoing apoptosis after treating the cells with chemotherapeutic and / or inhibitor **29** for the times indicated in Fig 5. Acridine orange has been shown to be taken up by both viable and non-viable cells and to intercalate into double stranded DNA and emit green fluorescence. Ethidium bromide, also a DNA intercalating agent, is normally taken up only by non-viable cells and emits a red fluorescence upon binding to DNA [27, 32]. Use of these dyes allows detection of the nuclear morphology of the cells. Fig 5 shows that exposure of DU145TXR cells to 15 μM P-gp inhibitor **29** for 24 hours, or 10 μM paclitaxel for 1 hour did not result in any observable signs of apoptosis, *i*.*e*. chromosomal and/or cellular fragmentation. Under these conditions, no non-viable cells were detected by ethidium bromide red fluorescence when compared to the DMSO-vehicle only controls (Fig 5, top three panels). The blue arrows point to examples of cells possessing highly organized nuclear morphologies typical of viable, non-apoptotic cells. Treatment of cells for 1 hour with the same concentration of paclitaxel in the presence of P-gp inhibitor **29** also did not appreciably increase the occurrence of cells displaying typical apoptotic morphologies (Fig 5, bottom left panel). In contrast however, relatively short 1-hour exposures to paclitaxel followed by treatment with P-gp inhibitor **29** for 23 hour dramatically increased the number of cells showing chromatin fragmentation (white arrows, bottom center panel) and non-viable dead cells with fragmented nuclei (yellow arrows, bottom center panel). The data strongly suggest that extended exposure of P-gp inhibitor **29** to the cancer cells after initial paclitaxel treatment resulted in much higher incidences of induced apoptosis. The extent of the induced apoptosis under the prolonged P-gp inhibitor treatments appeared to approach that resulting from a full 24 hour continuous exposure to paclitaxel.

### Cells that do not over-express P-gp are not significantly affected by extended treatment with P-gp inhibitor 29

**Fig 6:**
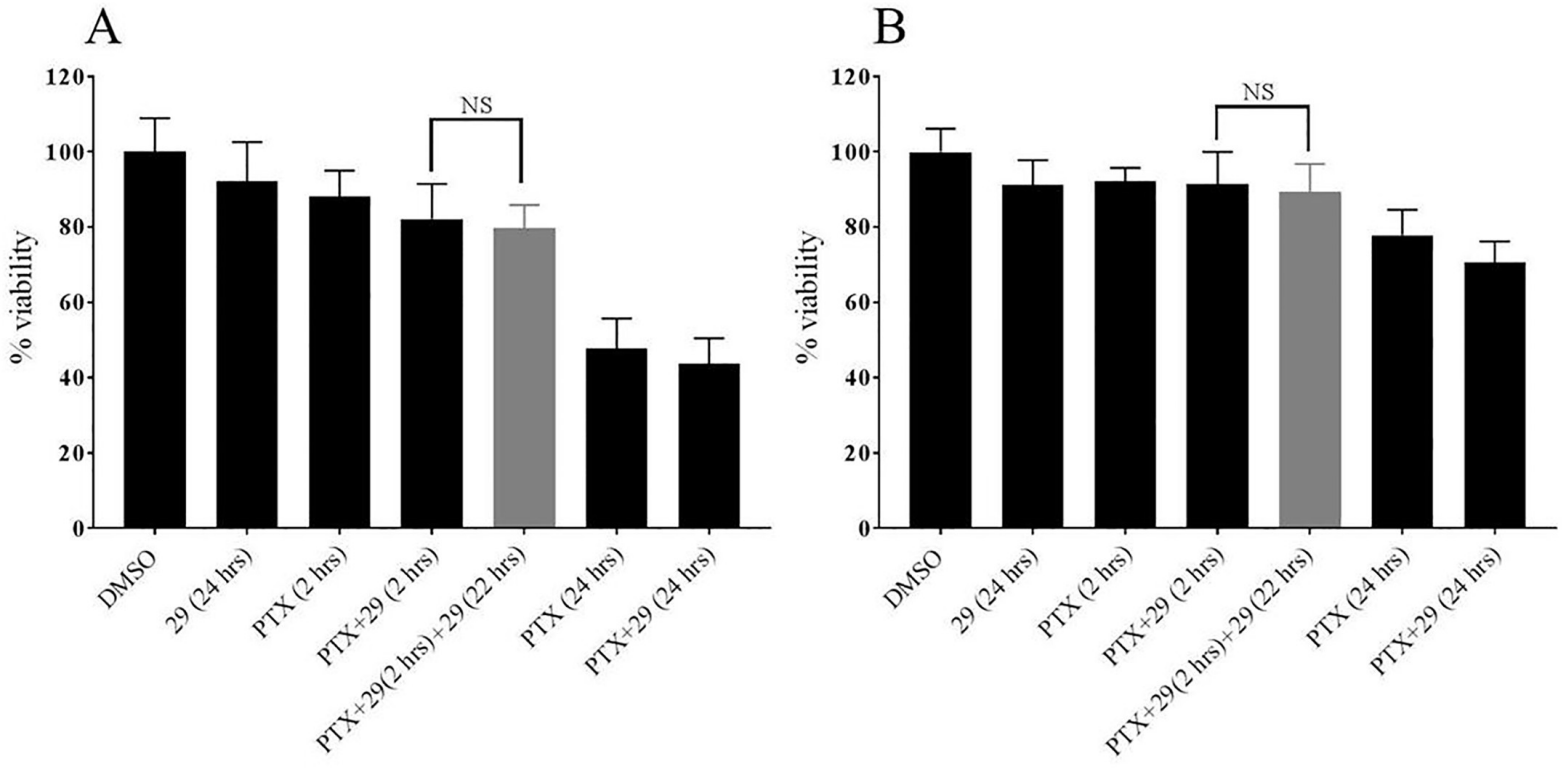
Extended treatments with P-glycoprotein inhibitor do not affect cells that do not over-express P-gp. In these experiments, cell viability was assessed with resazurin as described for Fig 2. **A**–DU145 cells were preloaded with paclitaxel (PTX) by incubation in the presence of 25 μM of P-gp inhibitor **29** unless otherwise indicated in the figure. After the preloading, cells were washed and media was replaced with complete media with the indicated additions. Control experiments included exposure of the cells to DMSO alone (0.5% final concentration), P-gp inhibitor **29** alone, paclitaxel alone, or in combinations and times indicated. Extended treatment with P-gp inhibitor **29** was carried out for 22 hours. **B**–Experimental protocols as in **A**, but using human lung fibroblast HFL-1 cells. For this experiment, the concentration of paclitaxel was 0.1 μM and that of compound **29** was 25 μM as indicated. The percentage of viable cells present at the end of the experiments were expressed as percentages of the DMSO- vehicle only control experiments. Data are expressed as averages +/- S.D. of duplicate experiments. “NS” = not significantly different. Measurements were made as described in Methods using a Cytation 5 imager.

For further evaluation of the new inhibitor for potential future development for clinical use it was of interest to determine whether the extended P-gp inhibitor **29** treatment after exposure to chemotherapeutics might increase cytotoxicity of the chemotherapeutics in cells that do not over-express P-gp. To this aim, experiments similar to those reported in Fig 2 were performed with the prostate cancer cell line, DU145 [20], that does not over-express P-glycoprotein [21] (Fig 6A) and with a relatively normal human lung fibroblast cell line, HFL-1[22] (Fig 6B), which also does not over-express P-gp. Cells were incubated with the either inhibitor **29** or paclitaxel alone with or without an extended P-gp inhibitor treatment in the combinations indicated in the figure. Inhibitor **29** was used here at the same concentrations used in the viability assays (Fig 2), while much lower concentrations of paclitaxel were required (0.1 μM). The lower chemotherapeutic concentrations were required because of the nearly 3 orders of magnitude increased paclitaxel sensitivity in these cell lines that do not over-express P-gp [15]. No significant differences in cell viabilities with either the DU145 or the HFL-1 cell lines were observed between paclitaxel plus inhibitor **29** treatments for 2 hours or the identical treatments followed by 22 hours of exposure to P-gp inhibitor **29** (Fig 6A and 6B). These experiments demonstrated that the extended P-gp inhibitor treatment did not increase the toxicity of chemotherapy treatment to non-P-gp overexpressing cells.

## Discussion

The balance between chemotherapy effectiveness and its associated toxicities to normal cells and tissues has been the principle limit to the clinical efficacy of cancer chemotherapies[33]. Contributing to this problem have been intrinsic or acquired resistances to chemotherapeutics that limit effectiveness on the targeted cancer cells. These resistances usually do not extend to normal cells and therefore do not protect the normal cells from the toxicities of the therapeutic agents (see for example [34]). The phenomenon of multidrug resistance caused by the over-expression of ABC transporter drug efflux pumps that have broad pump substrate specificities like P-glycoprotein has been particularly problematic due to the fact that these cellular defense proteins can limit the intracellular concentrations of drugs to sub-therapeutic levels specifically in the cancer cells [35, 36]. Especially troubling as a potential cause of cancer recurrence after apparently successful treatment is the cancer stem cell hypothesis [37–40] that posits that a subpopulation of cancer cells, well-protected by ABC transporter efflux pumps, may be responsible for the long-term growth and resistance of many cancers to chemo- and radiation therapies. The significant unmet medical need for targeted therapies against these MDR cancer cell defenses is therefore obvious.

Although the efficacy of the various P-gp inhibitors was proven to be a success in in vitro and some in vivo experiments [15, 16, 41, 42], P-gp inhibitors had shown only a limited success in clinical trial settings to date. We hypothesized that one reason for this could be due to the treatment strategy of clinical trials. In this study, we have demonstrated that continued inhibition of the drug transporter P-gp after relatively short exposures to chemotherapeutics in a P-gp overexpressing prostate cancer cell line, limited the efflux of the therapeutics from the cells. The results also indicated that in the presence of our novel P-gp inhibitor **29** after exposure and then removal of free chemotherapeutic, but in the continued presence of the inhibitor, significantly more chemotherapeutic drug accumulated in the cancer cells resulting in a higher effective steady state concentration of the drug inside the cells, which ultimately increased the efficacy of the treatment. The decreased chemotherapeutic efflux and resulting increased cellular concentration of chemotherapeutics in the presence of the P-glycoprotein inhibitor **29** was in turn associated with decreased cancer cell viability (Fig 2), decreased cancer cell survival (Fig 3), and decreased cancer cell motility (Fig 4), while simultaneously evidence of cell death *via* apoptosis increased (Fig 5). In stark contrast to these results, treatment of relatively normal cells that do not over-express P-glycoprotein or cancer cells that do not over-express P-glycoprotein did not show any signs of increased toxicities from the chemotherapeutic or the extended P-gp inhibitor treatments.

The results of these studies suggest that chemotherapy treatment of cancers that show a multidrug resistant phenotype due to over-expression of P-glycoprotein might become more efficacious if followed by an extended period of inhibition of the transporter. The lack of ability to expeditiously remove a chemotherapeutic from a patient after a short exposure complicates the translation to clinical applications of the work reported here in cell culture. It is interesting, however, to compare the pharmacokinetics of a known P-glycoprotein inhibitor, tariquidar, in human plasma after an intravenous injection of a 150 mg dose in a Phase I study [43] with the pharmacokinetics of doxorubicin in an unrelated study in women undergoing chemotherapy for breast cancer [44]. In the former study, the concentration of the P-gp inhibitor in blood plasma was at least 16-times its IC_50_ for P-glycoprotein 24 hours after administration (187 nM and 5 nM, respectively) even though the 24 hour level was 16 times below its maximal concentration (C_max_) of 3.06 μM [43]. In the latter study, the maximal plasma concentrations of doxorubicin in which a standard dosage of 60 mg / m^2^ body surface area was administered intravenously averaged 1.16 μM in the breast cancer patients [43] with an average concentration at 24 hours of about 51 nM (23 times lower than the C_max_). The half-times for tariquidar and doxorubicin in these studies averaged 33 and 49 hours, respectively. In these cases, the pharmacokinetics of each drug was significantly different with doxorubicin clearance slower than that of tariquidar. With respect to these results, translating the extended inhibitor treatments demonstrated by us here in cell culture to similar human clinical examples would require additional treatment(s) with the P-gp inhibitor to obtain the extended treatment advantages observed. Further, we cite several clinical studies that administered P-gp inhibitors along with chemotherapeutics [43, 45–49] and note that in all these studies, the doses of P-gp inhibitors were administered prior to or along with chemotherapeutics, without extended treatment of the P-gp inhibitor. These studies have demonstrated that the doses of P-gp inhibitors were well tolerated by the patients, but showed only marginally increased efficacy of chemotherapy. Based on our results in this study using a cell culture model, we hypothesize that addition of extra doses of a P-gp inhibitor (compound **29** was used here) after the initial chemotherapy treatments would likely increase the intracellular retention of chemotherapeutics in the patients’ cancer cells. This would likely increase the efficacy of treatment of these MDR cancers.

Despite these difficulties in implementing extended P-glycoprotein inhibitor treatments, we believe that extended inhibition of an ABC transporter responsible for an MDR phenotype during and after chemotherapy exposure and the associated increase in efficacy of treatment with the likely lack of associated increased toxicities is one worth considering.

## Supporting information

S1 File The original data used to create the figures for this manuscript can be found in the Supporting_Information.pdf file. (PDF)
