# Supplementary material for: Prolonged inhibition of P-glycoprotein after exposure to chemotherapeutics increases cell mortality in multidrug resistant cultured cancer cells
Source: PLoS One. 2019 Jun 7;14(6):e0217940. doi: 10.1371/journal.pone.0217940 (PMC6555590; doi:10.1371/journal.pone.0217940)
Supplement: S1 File — (PDF) [file pone.0217940.s001.pdf]

Data for Figure 1A

Trial 1

Time (min)	DNR + 29 followed bymedia only					
0	0	0	0	0	0	0
15	0.255509	0.301887	0.285631	0.233645	0.310759	0.223565
30	0.687909	0.822276	0.658879	0.484229	0.837975	0.740785
45	1.179869	1.221546	1.099299	0.952103	1.297468	0.99577
60	1.468731	1.459525	1.425234	1.29264	1.588608	1.351057
75	1.796307	1.786975	1.74007	1.620327	1.90443	1.688218
90	1.979154	2.01339	1.942757	1.815421	2.144304	1.944411
105	2.054199	2.101643	2.040888	1.973715	2.279114	2.103323

	DNR + 29 followed bymedia + 29					
0	0	0	0	0	0	0
15	0.273002	0.180596	0.240143	0.209723	0.256132	0.205562
30	0.671913	0.547049	0.442652	0.499152	0.520821	0.533857
45	0.890436	0.856224	0.718638	0.783493	0.722191	0.804716
60	1.076271	1.004091	0.928913	0.958168	0.93668	1.025998
75	1.309927	1.268849	1.140382	1.27247	1.186537	1.302902
90	1.419492	1.366452	1.344086	1.306388	1.216771	1.325272
105	1.49092	1.391584	1.408602	1.355003	1.346264	1.340992

Trial 2

Time (min)	DNR + 29 followed bymedia only					
0	0	0	0	0	0	0
15	0.103233	0.133725	0.121607	0.124867	0.159238	0.143793
30	0.261731	0.376194	0.318132	0.339737	0.361143	0.339655
45	0.408759	0.506613	0.47557	0.49164	0.529524	0.452069
60	0.594369	0.709037	0.654723	0.703308	0.69219	0.572069
75	0.708377	0.826598	0.793702	0.787264	0.811048	0.68
90	0.777546	0.889787	0.887803	0.86624	0.92	0.757931
105	0.847758	0.936811	0.935939	0.93063	0.986667	0.888276

	DNR + 29 followed bymedia + 29					
0	0	0	0	0	0	0
15	0.111351	0.00253	0.11194	0.099164	0.107969	0.080899
30	0.21009	0.146729	0.210377	0.237462	0.224385	0.241573
45	0.35027	0.307915	0.323383	0.415653	0.37679	0.417603
60	0.497297	0.404771	0.470149	0.510638	0.466765	0.538202
75	0.605045	0.518251	0.545842	0.596505	0.581711	0.648315
90	0.692613	0.567763	0.601635	0.671353	0.632024	0.667041
105	0.715315	0.615107	0.657783	0.721884	0.657363	0.717603

Data for Figure 1B

Trial 1

DNR + 29 followed by media + 29	DNR + 29 followed by media only	DNR only
10443	5185	5172
9510	5106	5101
10101	5285	5517
9098	4594	4072
9910	5350	3573
9123	4972	3504

Trial 1

DNR + 29 followed by media + 29	DNR + 29 followed by media only	DNR only
7337	3486	3039
8218	4840	3499
7959	4060	4561
7903	4761	4259
7018	5465	3817
7176	5258	4099

Data for Figure 2A

Trial 1

DMSO (0.5%)	29, 24 hrs (25 µM)	PTX 2 hrs (10 µM)	PTX+29, 2 hrs (25 µM)	PTX+29, 2 hrs -> 29, 22 hrs (25 µM)	PTX 24 hrs	PTX+29, 24 hrs (25 µM)
101	80	87	86	44	53	39
107	83	104	108	57	60	47
85	74	99	90	47	46	33
100	81	103	92	51	58	42
85	87					
76	78					
93	69					
	76					

Trial 2

DMSO (0.5%)	29, 24 hrs (25 µM)	PTX 2 hrs (10 µM)	PTX+29, 2 hrs (25 µM)	PTX+29, 2 hrs -> 29, 22 hrs (25 µM)	PTX 24 hrs	PTX+29, 24 hrs (25 µM)
107	85	92	76	53	55	49
97	91	72	68	48	50	44
110	78	73	76	48	46	35
110	84	90	69	48	40	34
111	109					
116	101					
	100					
	105					

Data for Figure 2B

Trial 1	DMSO	29 (24 hrs)	VIN (2 hrs)	VIN+29 (2 hrs)	VIN+29 (2 hrs) -> 29 (22 hrs)	VIN (24 hrs)	VIN+29 (24 hrs)
	101	80	97	83	40	49	37
	107	83	93	102	57	72	49
	85	74	84	85	40	55	33
	100	81	94	94	53	56	48
	85	87					
	76	78					
	93	69					

Trial 2	DMSO	29 (24 hrs)	VIN (2 hrs)	VIN+29 (2 hrs)	VIN+29 (2 hrs) -> 29 (22 hrs)	VIN (24 hrs)	VIN+29 (24 hrs)
	107	76	97	82	49	66	54
	97	85	90	81	45	57	39
	110	91	77	82	43	58	37
	110	78	71	77	48	66	32
	111	84					
	116	109					
		101					
		100					
		105					

Data for Figure 3B

Trial 1	DMSO	29 (24 hrs)	PTX (2 hrs)	PTX + 29 (2 hrs)	PTX + 29 (2 hrs) -> 29 (22 hrs)	PTX (24 hrs)
	107	141	110	113	21	5
	73	110	113	121	68	5
	68	107	84	110	34	8

Trial 2	DMSO	29 (24 hrs)	PTX (2 hrs)	PTX + 29 (2 hrs)	PTX + 29 (2 hrs) -> 29 (22 hrs)	PTX (24 hrs)
	102	105	113	110	13	8
	102	155	147	134	37	8
	147	139	126	131	21	10

Data for Figure 4B

Trial 1	DMSO	29 only (14 hrs)	PTX only (1 hr)	PTX+29 (1 hr)	PTX+29 (1 hr) ->29 (13 hrs)
	99	119	102	89	64
	97	115	106	95	39
	110	105	105	118	55
	80	114	105	116	45
	115	113	116	121	29
	99	109	89	94	47

Trial 2	DMSO	29 only (14 hrs)	PTX only (1 hr)	PTX+29 (1 hr)	PTX+29 (1 hr) ->29 (13 hrs)
	89	104	108	86	53
	100	106	96	90	57
	104	121	107	85	41
	101	95	106	74	50
	106	109	101	89	25
	100	103	98	88	42

Data for Figure 6A

	DMSO	29 (24 hrs)	PTX (2 hrs)	PTX+29 (2 hrs)	PTX+29 (2 hrs) -> 29 (22 hrs)	PTX (24 hrs)	PTX+29 (24 hrs)
Trial 1							
	105	85	98	88	89	50	48
	107	87	87	71	84	47	40
	92	84	83	81	69	45	42
	102	80	82	83	79	43	39
	101	81	84	74	79	41	35
	93	85	91	83	79	31	37

	DMSO	29 (24 hrs)	PTX (2 hrs)	PTX+29 (2 hrs)	PTX+29 (2 hrs) -> 29 (22 hrs)	PTX (24 hrs)	PTX+29 (24 hrs)
Trial 2							
	106	112	100	95	87	63	59
	116	106	96	100	82	56	45
	103	101	87	75	80	44	45
	97	101	88	88	83	51	49
	81	91	77	71	69	48	38
	97	92	83	77	77	53	48

Data for Figure 6B

	DMSO	29 (24 hrs)	PTX (2 hrs)	PTX+29 (2 hrs)	PTX+29 (2 hrs) -> 29 (22 hrs)	PTX (24 hrs)	PTX+29 (24 hrs)
Trial 1							
	104	96	97	91	91	85	74
	99	94	94	95	90	78	76
	100	92	92	90	89	84	75
	96	81	93	93	89	82	74
	103	89	91	86	84	73	66
	97	94	87	83	86	72	67

	DMSO	29 (24 hrs)	PTX (2 hrs)	PTX+29 (2 hrs)	PTX+29 (2 hrs) -> 29 (22 hrs)	PTX (24 hrs)	PTX+29 (24 hrs)
Trial 2							
	115	92	93	83	78	74	66
	99	88	93	108	99	74	73
	99	90	91	95	95	81	76
	94	91	99	105	99	90	76
	103	81	88	80	76	66	60
	90	106	88	88	96	75	64
